# Lower all-cause 30-day mortality during summer following foot and ankle fracture surgery: a Swedish perioperative register-based study

**DOI:** 10.2340/17453674.2025.44396

**Published:** 2025-08-07

**Authors:** Elin LUNDIN, Jon KARLSSON, Jan G JAKOBSSON

**Affiliations:** 1 Karolinska Institutet, Stockholm; 2 Sahlgrenska Academy, Gothenburg; 3 Department of Anaesthesia & Intensive Care, Karolinska Institutet, Stockholm, Sweden

## Abstract

**Background and purpose:**

An important quality indicator of perioperative care is the all-cause 30-day mortality. Little is known about early mortality after foot and ankle fracture repair. We aimed to assess the all-cause 30-day mortality associated with surgical repair of foot and ankle fractures in Sweden during 2017–2022 and its seasonal variation.

**Methods:**

Foot and ankle fracture patients aged ≥ 18 years registered in the Swedish Perioperative Quality Register (SPOR) between 2017 and June 30, 2022 were included in the analysis (n = 26,404). Patient characteristics, perioperative observations, and early mortality were collected. Seasonal variation was analyzed for summer, autumn, winter, and spring. Perioperative mortality rate and odds ratio (OR) are reported with 95% confidence intervals (CI).

**Results:**

The all-cause 30-day mortality rate was 58 of the 26,404 studied patients (0.22%, CI 0.17–0.28). There was no change in mortality rate over the study period including the COVID-19 pandemic year. Increased adjusted odds ratio (OR) for 30-day mortality was seen among the elderly, age > 80 years, OR 22 (CI 9.2–50), and those with low health status, ASA class 3–4, OR 4.2 (CI 2.3–7.9), while surgery during summer was associated with a lower adjusted OR 0.4 (CI 0.1–0.9).

**Conclusion:**

The all-cause 30-day mortality rate after foot and ankle fracture surgery in Sweden is reassuringly low with expected higher OR for mortality associated with age and health status, while surgery during summer months was associated with lower mortality.

All-cause 30-day mortality is one well-accepted quality indicator of perioperative care providing the possibility of making comparisons between healthcare systems [1]. The incidence of ankle fractures has a profound seasonal variation in the Nordic countries [2-4], and most fractures occur during wintertime. The seasonal variability in surgical outcome and all-cause 30-day mortality associated with foot and ankle fracture surgery is, however, not well studied. A recent study on seasonal variation in surgical outcome showed an increase in surgical site infections and mortality during winter, which is more pronounced in emergency procedures [5]. There is no recent study that has assessed the all-cause 30-day mortality among patients undergoing foot and ankle fracture repair and the impact of season. Whether the all-cause 30-day mortality is higher during the winter months in Sweden and, further, the impact of age and health status (i.e., ASA class), are unknown.

We aimed to assess the all-cause 30-day mortality associated with surgical repair of foot and ankle fractures in Sweden during 2017–2022 and its seasonal variation. The hypothesis was that 30-day mortality should be higher during winter after adjusting for age and ASA class.

## Methods

### Study design

This is a register-based study, based on data from the SPOR register [6,7]. It was designed as a national, observational, retrospective, quality register study with no additional data collection. The included study population comprised all foot and ankle fractures undergoing surgery (ICD code NHJ00-99) in patients aged ≥ 18 years registered in the national Swedish perioperative quality register (SPOR) [7] between January 1, 2017, and June 30, 2022 (n = 26,404). In patients with surgery on several occasions, only the last procedure was analyzed for all-cause mortality. The manuscript adheres to the STROBE guidelines for cohort studies.

### Data collected

SPOR [6] is a national quality register collecting perioperative data started in 2011. It has an increasing coverage, and a vast majority of hospitals are today reporting to the register. 71 base variables including patient data, time stamps, codes for the surgical procedure and diagnoses, and anesthetic technique. Outcomes and quality parameters (e.g., perioperative bleeding, patient body temperature, postoperative pain, nausea/vomiting, and selected vital parameters) are also reported. It cross-references with the Swedish death register. It has been validated and found to have good agreement between local and central databases [7].

Age, sex (male/female), ASA class (1–5), duration of anesthesia, surgery and recovery room stay, compliance with WHO safe surgical checklist, and all-cause 30-day mortality were collected.

The primary outcome was all-cause 30-day mortality rate as defined by the revised Utstein definition [8].

Exposure was the 4 seasons, winter, spring, summer, and autumn, in line with the hypothesis of increased risk of 30-day mortality during winter months

### Categorization of data

The study population was divided into 4 groups based on the season in which surgery was performed: winter (December–February), spring (March–May), summer (June–August), or autumn (September–November).

Age was categorized into 3 groups: 18–65 years, 66–80 years, and > 80 years of age. ASA was divided into classes 1–5 in accordance with ASA definitions [9] and further classified as low-risk (ASA 1–2) and high-risk (ASA 3–5).

### Statistics

Data was presented for the entire study population and seasonally for the time studied (2017–2022), and no power analysis was performed. Continuous numerical data, such as age and time events, was presented as mean and standard deviation (SD). Categorical variables, such as sex and ASA class, were presented as numbers and percentages (%). To determine whether continuous numerical data was normally distributed, histograms were made in Excel (Microsoft Corp, Redmond, WA, USA) for ocular assessment. Comparisons of continuous normally distributed numerical data between the 4 seasons and deceased and alive were performed with a parametric test, one-way ANOVA with Tukey’s post-hoc analysis. A non-parametric test, Mann–Whitney U-test, was performed for skewed data. Differences in proportions between the 4 seasons and deceased and alive were analyzed with a chi-square test and Fisher’s exact test.

Further comparisons of 30-day mortality between seasons were conducted using binary logistical regression and presented as odds ratios (OR) together with 95% confidence intervals (CI). The OR for all cause 30-day mortality was analyzed adjusted for year and seasons of surgery, age class, ASA class, and sex.

Primary outcome 30-day mortality was available for all patients. Missing data for patients’ characteristics and season were assessed as missing completely at random (MCAR), thus analysis was performed on available information.

A P value < 0.05 was considered statistically significant. All data derived from the SPOR register was initially handled in Microsoft Excel (2024, version 16.83). Statistical analysis was performed using the SPSS statistical program (IBM SPSS Statistics, version 29, Armonk, NY, USA).

### Ethics, data sharing, funding, and disclosures

The study was approved by the Ethical Review Board Protocol number 2022-02521-02, Uppsala Department 2 of Medicine. This study is based on secondary data analysis, so new data is not being measured or produced; data is available via the SPOR register. The study was supported by the Department of Anaesthesia at Danderyd Hospital and the Institute of Clinical Sciences, Karolinska Institutet at Danderyd Hospital. The authors received no funding for this work. Jan Jakobsson is a paid safety physician for AstraZeneca. Authors report no further conflict of interests related to current manuscript. Complete disclosure of interest forms according to ICMJE are available on the article page, doi: 10.2340/17453674.2025.44396

## Results

**Figure 1:**
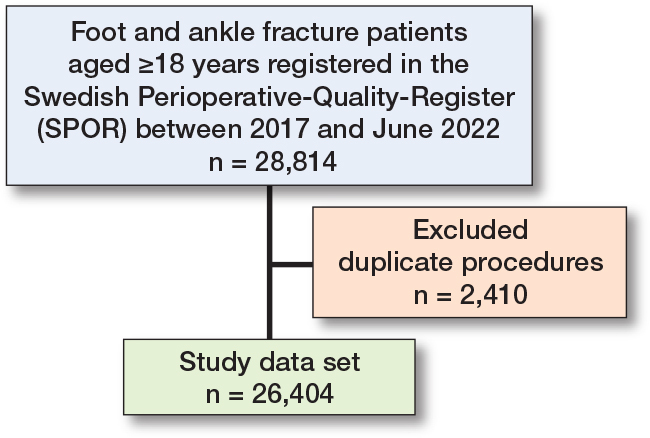
Flowchart describing patients included in study dataset.

The study cohort consists of 26,404 patients’ last surgical procedures for foot and ankle fracture. In all 2,410 procedures were duplicates and subsequently excluded from the analysis (Figure 1). Missing information regarding patients’ characteristics are sex in 151 patients and ASA class in 813 patients. The cohort divided by year and season shows an increasing number of procedures, 2,017 to 2,019 and decline in 2020, the COVID-pandemic year, and an increase in 2021 as well as in the first 6 months 2022 (Table 1).

**Table 1:** Patients having surgery for foot and ankle fracture over the 5.5 years studied, 2017–June 30, 2022. Values are number of patients deceased/alive

Season	2017	2018	2019	2020	2021	2022 ^a^	Total
Winter	5/1,106	3/1,347	4/1,708	4/1,186	4/1,902	2/1,248	22/8,597
Spring	2/774	5/1,264	2/1,135	3/1,080	3/1,070	2/1,364	17/6,687
Summer	1/839	2/994	0/1,247	1/1,341	2/1,318	0/412	6/6,151
Autumn	1/819	2/901	4/1,042	4/1,130	2/1,119	–	13/5,011
Total	9/3,538	12/4,506	10/5,132	12/4,737	11/5,409	4/3,024	58/26,346

a 6-month data, January 1–June 30.

There was a female predominance, 57%, and adults with ASA class 1–2 were the most common (Table 2). Overall, ASA class 2 was the most common (n = 11,581), followed by ASA class 1 (n = 10,241). The most common ASA class in the 18–65 years age group was ASA class 1. In patients between 66 and 80 years, ASA class 2 was the most common, and in the over 80 years patient group, ASA class 3 was most frequent (Table 3).

**Table 2:** Patients’ characteristics and perioperative data for patients having surgery for foot and ankle fracture during the period 2017–June 30, 2022. Values are count unless otherwise specified

Sex	
female	14,980
male	11,273
Mean age (SD)	53 (18)
Age category	
18–65	18,921
66–80	6,018
> 80	1,465
ASA class	
1	10,241
2	11,581
3	3,612
4	157
missing	813
ASA low (1–2)	21,822
ASA high (3–4)	3,769

SD = standard deviation, ASA class = American Society of Anesthesiologists physical status classification system

**Table 3:** Relationship between age and ASA class in the study population and proportion of dead. Values are group count and percentage of deceased

Age group	ASA 1	ASA 2	ASA 3	ASA 4
18–65	9,342 (0.01)	7,625 (0.04)	1,335 (0.30)	47 (2.2)
66–80	863 (0)	3,366 (0.09)	1,528 (0.39)	64 (12)
> 80	36 (0)	590 (1.9)	749 (2.0)	46 (15)

ASA: see Table 2.

### Mortality

**Figure 2:**
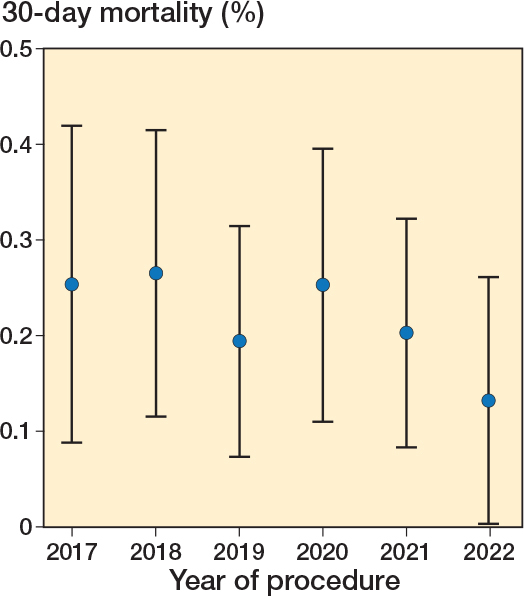
Annual all-cause 30-day mortality rates over the 6-year study period 2017–June 30, 2022 with 95% confidence intervals.

**Figure 3:**
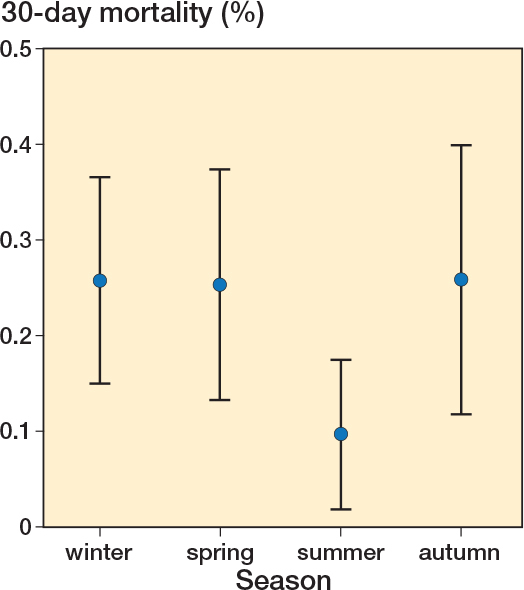
All-cause 30-day mortality rates per season, winter, spring, summer, and autumn with 95% confidence intervals.

Overall, 58 patients died within 30 days out of the 26,404 included in the analysis, i.e., 0.22% (CI 0.17–0.28) (see Table 1). There was minor variability in annual mortality rate (Figure 2) and for the entire cohort over the 4 seasons (Figure 3).

The logistic regression assessing all-cause 30-day mortality adjusted for patients’ characteristics’, sex, age, ASA class, and year as well as season of procedure showed an increased OR for high ASA class and even more for age > 80.

There were only minor differences in the OR for 30-day mortality over the study period, even including 2020, the COVID-19 pandemic year, which did not show any significant difference compared with the year before or after. The summer period was associate with a statistically significant lower odds ratio compared with winter (Table 4).There was a profound increase in mortality in ASA 4, especially among elderly patients (see Table 3).

**Table 4:** Logistic regression assessing adjusted odds ratio (OR) and 95% confidence interval (CI) for all-cause 30-day mortality associated with foot and ankle fracture surgery in Sweden 2017–2022, based on SPOR register data, unadjusted and adjusted for covariates

Item	OR (CI)
Age (ref. 18–65)	
66–80	3.7 (1.6–8.6)
> 80	22 (9.2–50)
ASA class (3–4 vs 1–2)	4.2 (2.3–7.9)
Sex (male vs female)	1.1 (0.6–2.0)
Year of surgery (ref. 2017)	
2018	1.0 (0.4–2.4)
2019	0.7 (0.3–1.8)
2020	1.0 (0.4–2.4)
2021	0.8 (0.3–1.9)
2022	0.5 (0.2–1.7)
Season (ref. Winter)	
Spring	1.0 (0.5–1.9)
Summer	0.4 (0.1–0.9)
Autumn	0.8 (0.4–1.7)

ASA class: see Table 2.

## Discussion

The purpose of this national, quality register study was to investigate seasonal differences, and whether there is a high risk for all-cause 30-day mortality during winter, associated with surgical repair of foot and ankle fractures in Sweden. The main findings were that the 30-day mortality rate in the total study population was 0.22%, and with merely minor differences over the 5.5-year study period, but when adjusting for sex, age, and ASA class, summer was associated with lower OR for all cause 30-day mortality. Advancing age and increasing ASA class were associated with significantly higher OR for 30-day mortality, as expected, while sex did not impact the OR for a fatal outcome in the adjusted analysis.

### Perioperative mortality

Perioperative mortality for foot and ankle fracture repair was found to be reassuringly low, while advancing age and higher ASA class were associated with increasing early mortality, as expected. Our previous SPOR-based studies investigating perioperative mortality following hip [10] and wrist fracture repair [11] showed a similar association between age and ASA class in relation to early mortality. However, in contrast to our study assessing foot and ankle fracture repair, females had an increasing risk of early mortality associated with wrist surgery [11], while surgery of hip fractures showed a higher risk among male patients [10]. In comparison with the present study, the 30-day mortality rate among patients undergoing hip and wrist fracture repair was either much higher (7.7%) or far lower (0.08%), respectively, placing early mortality following foot and ankle fracture repair in Sweden in between hip and wrist fracture repair [10,11].

We could not identify previous studies assessing early, all-cause 30-day mortality rate, following foot and ankle fracture repair in Sweden or the Nordic countries, and studies reporting this particular outcome are somewhat limited. In a study from Belmont et al. [12] based on the American College of Surgeons National Surgical Quality Improvement Program (ACS-NSQIP) database covering all ankle fractures requiring surgery in patients aged 18 years or more, between 2006 and 2011, a 30-day mortality rate of 0.30% was reported. Belmont et al. reported a more in-depth description of medical comorbidities and complications, making it possible for them to conclude an increasing risk of mortality after ankle fracture surgery among patients diagnosed with chronic obstructive pulmonary disease. Our study revealed an increased risk for early mortality among patients with an ASA class 2 or higher, which reflects a higher burden of medical comorbidities and systemic disease, including COPD, among others. Xu et al. [13] conducted a study based on the same American register as Belmont et al. including all patients aged 18 years or more, undergoing lower extremity repair of hip, knee, and ankle fractures between 2010 and 2019. They reported a 30-day mortality rate of 0.52% following surgery for foot and ankle fractures, which may indicate a higher mortality rate in the USA. We reported a slightly lower 30-day mortality rate.

### Seasonal variation in incidence of foot and ankle fracture

Previous studies from Sweden and the Nordic countries have concluded that there is a seasonal variation in the incidence of foot and ankle fractures [2-4]. In a Japanese study by Ogawa et al. [14] the seasonal impact on in-hospital mortality after hip fracture surgery was addressed. They concluded that there was a significantly higher mortality during fall and winter largely due to an increased incidence of respiratory infections. A recent meta-analysis from the UK addressing seasonal variations in patient outcomes in perioperative care provided evidence for an increased risk of postoperative surgical site infections in the summer and an increased risk of worse outcomes after surgery in the winter [5]. Though less studied, the seasonal variability results of our study appear to be in line with previous studies. Since we did not address medical comorbidities and peri- and postoperative complications, we can only speculate whether the lower 30-day mortality rate seen during summer is because of a lower rate of respiratory diseases or other seasonal infections, or because the seasonal subgroups might have different medical risk profiles.

### Patient characteristics

The patient characteristics of our study population align with previous literature. The Swedish epidemiological study by Rydberg et al. [2] spans the same period and presents almost the same mean age and sex distribution. In a previous study from Denmark [2-4], the sex distribution of the study population was similar to our study, but the mean age was more than 10 years lower. That may be because they included patients aged ≥0 years. The patient population in the study by Belmont et al. [12] assessing early mortality after ankle fracture repair is also notably similar to our study in terms of age, sex, and ASA class. However, Belmont et al. also presented information on comorbidities, revealing diabetes and morbid obesity as notable comorbidities. When comparing the characteristics between the seasons in our study, patients having surgery during summer were significantly younger. However, it is questionable whether this merely 2-year mean age difference is clinically relevant. Patients undergoing foot and ankle fracture repair during summer also had a significantly higher proportion of low-risk ASA, but similarly, the clinical significance of the 2% difference can be argued. Our patients’ characteristics are also in line with the Danish register-based study for the later period studied 1997–2018 [15].

### Strengths

A main advantage of the SPOR is its inclusion of variables from the whole perioperative care process and not only collecting data primarily concerning the anesthesia process or specific surgical procedures [16]. A recent validity study by Holmström et al. [7] also found the SPOR to be reliable and accurate overall, revealing good agreement between local data and the SPOR.

### Limitations

The continuous expansion of the relatively new SPOR register must be acknowledged. The number of participating hospitals in the register has increased over time but the SPOR’s coverage rate is still not 100% [7]. However, with regards to publicly funded hospitals caring for emergent patients the coverage rate is today assessed as acceptable and its use is supported [16,17]. As foot and ankle fractures surgery are emergent procedures we do not see coverage having any significant impact on our results. Missing data was overall minor and all random, thus it should not, in this huge dataset, have had any significant impact on our main results. The SPOR lacks some variables, such as detailed information on patients’ characteristics, comorbidities, and the clinical course after discharge from the recovery room. Finally, the register does not provide any information regarding anesthesia and fracture mechanism and severity, indication for surgery, and whether the foot and ankle fracture is concomitant with any other injury.

These factors may have contributed to the outcome, and we have not reviewed causes of death, comorbidities, and complications after the end of recovery care in detail, as this information is not available in the register.

### Conclusions

The perioperative 30-day mortality rate following foot and ankle fracture repair in Sweden was 0.22%. The summer season was associated with significantly lower odds for 30-day mortality. High age, especially above 80 years, and high ASA class was expectedly associated with higher odds of early mortality, when adjusted for sex, age and ASA class.

*In perspective,* further efforts to improve perioperative care, preoperative optimization, and postoperative observation should be considered for above 80-year-old ASA 3–4 patients.

## Notes

Concept and protocol: JJ and EL. Data handling and analysis: EL and JJ (support by Fredrik K Johansson, KI statistician). Writing manuscript: EL and JJ. Manuscript review and input: JK.

Fredrik K Johansson has supported all statistical analyses. Klara Bergmark, head of department anaesthesia & intensive care, has supported with working hours and mental support.

Handling co-editors: Bart A Swierstra and Robin Christensen

*Acta* thanks Harri Juhani Pakarinen and Tim Schepers for help with peer review of this manuscript.

## References

[1] Clench-Aas J, Helgeland J, Dimoski T, Gulbrandsen P, Hofoss D, Holmboe O, et al. Methodological development and evaluation of 30-day mortality as quality indicator for Norwegian hospitals [Internet]. Oslo, Norway: Knowledge Centre for the Health Services at the Norwegian Institute of Public Health (NIPH); 2005 Sep. Report from Norwegian Knowledge Centre for the Health Services (NOKC) No. 04-2005. PMID: 29319970. 29319970

[2] Rydberg E M, Wennergren D, Stigevall C, Ekelund J, Möller M. Epidemiology of more than 50,000 ankle fractures in the Swedish Fracture Register during a period of 10 years. J Orthop Surg Res 2023; 18(1): 79. doi: 10.1186/s13018-023-03558-2. 36721256 PMC9887758

[3] Elsoe R, Ostgaard S E, Larsen P. Population-based epidemiology of 9767 ankle fractures. Foot Ankle Surg 2018; 24(1): 34-9. doi: 10.1016/j.fas.2016.11.002. 29413771

[4] Happonen V, Kröger H, Kuismin M, Sund R. Ankle fractures in Finland: 118,929 operatively treated between 1987 and 2019. Acta Orthop 2022; 93: 327-33. doi: 10.2340/17453674.2022.2071. 35147707 PMC8833737

[5] Spencer E, Berry M, Martin P, Rojas-Garcia A, Moonesinghe S R. Seasonality in surgical outcome data: a systematic review and narrative synthesis. Br J Anaesth 2022; 128(2): 321-32. doi: 10.1016/j.bja.2021.10.043. Epub 2021 Dec 4. PMID: 34872715. 34872715

[6] SPOR – Swedish Perioperativt Register. Available from: https://spor.se/ (accessed July 26, 2025).

[7] Holmström B, Enlund G, Spetz P, Frostell C. The Swedish Perioperative Register: description, validation of data mapping and utility. Acta Anaesthesiol Scand 2023; 67(2): 233-9. doi: 10.1111/aas.14174. 36424870 PMC10108284

[8] Davies J I, Gelb A W, Gore-Booth J, Martin J, Mellin-Olsen J, Åkerman C, et al. Global surgery, obstetric, and anaesthesia indicator definitions and reporting: an Utstein consensus report. PLoS Med 2021; 18(8): e1003749. doi: 10.1371/journal.pmed.1003749. 34415914 PMC8415575

[9] American Society of Anesthesiologists. Statement on ASA Physical Status Classification System. Available from: https://www.asahq.org/standards-and-practice-parameters/statement-on-asa-physical-status-classification-system (accessed July 26, 2025).

[10] Gremillet C, Jakobsson J G. Acute hip fracture surgery anaesthetic technique and 30-day mortality in Sweden 2016 and 2017: a retrospective register study. F1000Res 2018; 7:1009. doi: 10.12688/f1000research.15363.2. 30210789 PMC6107981

[11] Sellbrant I, Nellgård B, Karlsson J, Albert J, Jakobsson J G. Anaesthesia practice, quality indices including all-cause 30-day mortality associate to wrist fracture repositioning and surgery in Sweden: a perioperative register-based study 2018–2021. Acta Anaesthesiol Scand 2024; 68(3): 402-9. doi: 10.1111/aas.14358. 37952557

[12] Belmont P J Jr, Davey S, Rensing N, Bader J O, Waterman B R, Orr J D. Patient-based and surgical risk factors for 30-day postoperative complications and mortality after ankle fracture fixation. J Orthop Trauma 2015; 29(12): e476-82. doi: 10.1097/BOT.0000000000000328. 25785357

[13] Xu A L, Raad M, Sotsky R B, Hughes A J, Aiyer A A. Comparative risk stratification for prediction of early postoperative morbidity and mortality after open fixation of periarticular lower extremity fractures. J Clin Orthop Trauma 2022; 31:101940. doi: 10.1016/j.jcot.2022.101940. 35865328 PMC9294326

[14] Ogawa T, Yoshii T, Higuchi M, Morishita S, Fushimi K, Fujiwara T, et al. Seasonality of mortality and in-hospital complications in hip fracture surgery: retrospective cohort research using a nationwide inpatient database. Geriatr Gerontol Int 2021; 21(5): 398-403. doi: 10.1111/ggi.14153. 33768645

[15] Gundtoft P H, Pedersen A B, Viberg B. Incidence, treatment, and mortality of ankle fractures: a Danish population-based cohort study. Acta Orthop 2025; 96: 203-8. doi: 10.2340/17453674.2025.43006. 40029096 PMC11868812

[16] Jammer I, Brandsborg B. How to improve perioperative pathways for the patient and society. Acta Anaesthesiol Scand 2023; 67(2): 126-7. doi: 10.1111/aas.14192. 36583646

[17] Kvåle R, Möller M H, Porkkala T, Varpula T, Enlund G, Engerstrôm L, et al. The Nordic perioperative and intensive care registries: collaboration and research possibilities. Acta Anaesthesiol Scand 2023; 67(7): 972-8. doi: 10.1111/aas.14255. 37096912

